# Death under Nitrous Oxide

**Published:** 1901-03

**Authors:** 


### DEATH UNDER NITROUS OXIDE.

A STRANGE and, luckily, rare incident has lately occurred in the practice of Mr. H. D. Griffiths, of Newport. A young and apparently healthy man consulted Mr. Griffiths, who is an L.D.S.I. and an old-established practitioner, with respect to the extraction of some teeth, and it was decided to put the patient under an anæsthetic. Nitrous oxide gas, followed by ether, was administered by Mr. Griffiths, who was assisted in the operation by a nurse who had been some months in his employment. The report does not say whether she was a certificated nurse, but presumably she was a woman of experience. The patient took the anæsthetic well, and eleven teeth were extracted,—the operation lasting in all about three minutes,—the nurse using a Turkish sponge to absorb blood. The operation had been successfully completed and the young man had not fully regained consciousness, when, after a deep inspiration, it was seen that breathing had ceased. Mr. Griffiths resorted to artificial respiration and immediately sent for his neighbor, Dr. Stewart Vines, who came in about five minutes, and joined Mr. Griffiths in his efforts to restore animation by injecting stimulants and by every means known to them. All was in vain, as the patient never regained consciousness. The inquest revealed the cause of death, as the sponge which had been used by the nurse was found firmly impacted in the air-passages. The nurse had quite forgotten that she had left the sponge in the mouth of the patient, who with a deep inspiration had drawn it down beyond the point from which it could be observed, as Mr. Griffiths had examined the throat to see if there was any obstruction.

The coroner, in summing up, expressed sympathy with the deceased and his relatives and also with Mr. Griffiths, to whom he imputed no blame, and the jury brought in a verdict of "death by misadventure."

We unite with the coroner in his expression of sympathy, but we cannot but think that this sad case teaches us all the lesson we are so apt to forget,—that our operations are conducted over a cavity, the importance of which cannot be exaggerated, namely the glottis, into which any object, however trifling, falling, may cause death. Instances are not wanting in which a tooth, the broken beak of a forceps, and other objects have fallen with fatal results; while in many cases these results have only been obviated with great difficulty. Even in such a simple case as touching a tooth with a stick of nitrate of silver, insecurely fastened, the stick has slipped down, doing considerable damage. The most important factor in counteracting untoward accidents is to recognize the cause and remove it. In the present case the cause was not discovered until too late. Prevention is better than cure, and the fauces should be guarded by every means in our power. In extraction, the finger or thumb should be always interposed between it and the tooth; in applying medicaments to the teeth, care should be taken that they are firmly held by the carrier; and in the case of mouth-props and sponges, they should never be used without being attached to something which will render them easily recoverable. If the object has gone out of sight, prompt action is of vital importance, the first being that of inversion of the patient, followed by tracheotomy if necessary.

While regretting this sad incident, if it impresses these lessons upon our minds it will at least have been to us of some value.—*Editorial in British Journal of Dental Science.*

